# Development, implementation and first insights of a time- and location-independent longitudinal postgraduate curriculum in emergency medicine

**DOI:** 10.3205/zma001190

**Published:** 2018-11-15

**Authors:** Thomas C. Sauter, Aristomenis Exadaktylos, Gert Krummrey, Beat Lehmann, Monika Brodmann-Maeder, Wolf E. Hautz

**Affiliations:** 1 University of Bern, Bern University Hospital, Inselspital, Department of Emergency Medicine, Bern, Switzerland

**Keywords:** curriculum development, emergency medicine, medical education, Switzerland

## Abstract

**Introduction, background and context: **There have been few reports on the implementation of a structured curriculum for emergency medicine, as emergency medicine is not yet an established medical specialty for training in many European countries, including Switzerland and Germany. Because of the non-plannable workload in the emergency setting, common training approaches are often difficult to implement. Need-assessments of emergency medicine trainees commonly identify a need for interactive, time-independent ways of learning that integrate modern forms of knowledge transfer.

**Methods: **In the present study, we assess the local needs of emergency medicine specialists and trainees for a curriculum in emergency medicine and elaborate possible solutions for the implementation of this curriculum, taking into account the special needs in a highly dynamic, unplannable environment, such as an interdisciplinary emergency department.

**Results:** We describe the development of the emergency medicine curriculum on the basis of the six steps proposed by Kern for curriculum development in medical education, as well as the implementation, lessons learned and interval evaluation.

**Conclusions: **The combination of multiple teaching formats, ranging from time- and location-independent solutions such as podcasted lectures to simulation-based training sessions, as well as small-group workshops and skill training sessions, might be a valuable approach to implementing a state-of-the-art curriculum in a busy emergency department.

## Zusammenfassung

**Einleitung und Hintergrund: **Bis dato gab es nur wenige Berichte über die Implementierung eines strukturierten Curriculums für Notfallmedizin, da sich die Notfallmedizin als solche in vielen europäischen Ländern, einschließlich der Schweiz und Deutschland, im Bereich der Ausbildung noch nicht als medizinische Fachrichtung etabliert hat. Aufgrund der nicht planbaren Arbeitsbelastung im Setting der Notfallmedizin sind gängige Ausbildungsmethoden oftmals nur schwer umzusetzen. Bedarfsanalysen, bei denen Auszubildende im Bereich der Notfallmedizin befragt werden, identifizieren häufig den Bedarf an interaktiven, zeitunabhängigen Lernformen, die moderne Methoden des Wissensaustauschs integrieren.

**Methoden: **In der vorliegenden Studie werden der Bedarf der Notfallmedizinern und Auszubildenden vor Ort bezüglich eines Curriculums für Notfallmedizin bestimmt. Unter Berücksichtigung der besonderen Anforderungen in einem derart hochdynamischen, nicht planbaren Setting, wie eine interdisziplinäre Notaufnahme das ist, werden zudem mögliche Lösungsansätze für die Implementierung dieses Curriculums ausgearbeitet.

**Ergebnisse: **Dieses Paper beschreibt die Entwicklung des Notfallmedizincurriculums auf Basis der sechs Schritte für die Entwicklung von Curricula in der medizinischen Ausbildung nach Kern, die Implementierung und die daraus gewonnenen Erkenntnisse, sowie eine Intervallevaluierung.

**Schlussfolgerung: **Die Kombination verschiedener Lehrformen, von zeit- und ortsunabhängigen Lösungen, wie aufgezeichnete und online zugängliche Vorlesungen, bis zu simulationsbasierten Trainingseinheiteneinheiten sowie Kleingruppenworkshops und Skillstrainingseinheiten, könnte ein wichtiger Ansatz für die Implementierung eines hochmodernen Curriculums in einer geschäftigen Notaufnahme sein.

## Introduction

In many European countries, including Switzerland and Germany, clinical emergency medicine has not yet been established as an independent medical speciality with specific training [1]. In 2009, a certificate of clinical emergency medicine (Fähigkeitsausweis Klinische Notfallmedizin) was introduced by the Swiss Society of Emergency Medicine (SGNOR). A formal description of objectives and requirements, based on the curriculum of the European Society of Emergency Medicine (EUSEM) [2], has been published by the SGNOR [3] and serves as an outcome framework and basis for outcome-based education [4], [5], in accordance with current methodological standards in medical education [6]. Candidates who meet the predefined standards finally have to pass an examination, which is offered annually [3]. 

However, in many cases, training for physicians interested in emergency medicine and doctors working in emergency departments (ED) is still more or less a question of “learning on the job” and a structured curriculum for emergency medicine is rarely implemented. This is especially the case in smaller hospitals in Switzerland with limited training resources. In contrast to established international curricula for both medical students and residents, there is no national Swiss curriculum in emergency medicine [7], [8].

An obvious practical problem in emergency medicine education, as with other specialties with shift work [9], is whether it is possible to reach medical staff for specific training sessions. No matter which specific time frame is selected, only a small percentage of staff can be reached in systems with shift work. Due to the non-plannable workload in the emergency setting, even the staff on duty might not be able to attend training sessions. This is even more the case in smaller hospitals with limited staff resources.

Consequently, needs-assessments of emergency medicine trainees commonly identify a need for interactive, time-independent ways of learning, which integrate with modern forms of knowledge transfer, e.g. video-based learning formats [10], [11].

In the present study, we aimed to assess the needs of specialists in emergency medicine and trainees for a curriculum in emergency medicine and to consider possible approaches to implementing this curriculum in our emergency department - taking into account the special needs in this highly dynamic, unplannable environment.

## Methods and description of project

The development of the clinical emergency medicine curriculum is based on Kern’s 6 steps for curriculum development in medical education [12].

- The first step was identification of the problems in our setting.
- The second step was assessment of general needs and target needs.
- The third step was definition of goals and objectives.
- The fourth step consisted of allocation of educational strategies to each topic.
- The fifth step consisted of implementation.
- The sixth step contained feedback and evaluation measurements.

### Step 1: Setting

The ED of the Inselspital, University Hospital, Bern, Switzerland is a self-contained unit, a Level 1 trauma centre, with about 45,000 emergency consultations per year [13]. The ED is not only responsible for emergency medicine training in the medical school of Bern University, but also training residents whose ambition it is to be awarded the Certificate of Clinical Emergency Medicine. 

#### Step 2: Needs-assessment 

The needs-assessment at our department was conducted in three steps: Firstly, a literature search was undertaken to identify curricula for clinical emergency medicine that are available in Europe and Switzerland, as well as the curricular demands of the societies for emergency medicine. In the second part of the needs-assessment, all physicians working in the ED were surveyed with a structured questionnaire on their learning needs (questionnaire available upon request from the authors). In a third step, the identified curricula were assessed by local consultants, in order to tailor the content to the local needs.

#### Step 3 and 4: Definition of learning goals and allocation of educational strategies and formats

Under the guidance of two educational experts, appropriate teaching methods were selected for each theme (e.g. skill training, simulation-based team training, lectures). Objectives that were relevant in our setting were allocated to an appropriate teaching method. Finally, a responsible consultant was assigned to each topic.

#### Step 5: General implementation

The lecture-based formats are to coincide with shift changes, so that participation is mandatory for all staff on duty. The topics of the podcasted lectures are repeated and updated after one year and are thus continually modified. During the year of residence training, participation in all simulation-based training formats and skill training is mandatory for all residents in the department.

#### Step 6: Evaluation

Acceptance of the curriculum was monitored during and immediately after implementation, on the basis of open discussions with ED physicians during regular meetings. 6 months after the start of implementation, a structured online survey of all physicians at the ED was carried out as follow-up to the initial survey.

For the two surveys performed before and after implementation of the structured curriculum, a 5 point anchored Likert scale was used - ranging from 1 (completely unsatisfied) to 5 (very satisfied). All results are reported as means and standard deviation.

One possibility would be to report the data as median and inter-quartile range because they are scaled as ordinates and there has been much discussion about the advantages and disadvantages in the literature ([14]. We decided to use mean and standard deviation because these provide higher resolution and thus more information to the reader. Because of the relatively small numbers of physicians in our department, the results of this exploratory study are presented descriptively and without formal testing. Effects on interprofessional learning and collaboration – together with patient outcomes – are presented in a separate study [15].

## Results

### Step 1: Problem identification

As described above, our department is involved in training students in emergency medicine at Bern University Medical School and at the training centre for residents for the Certificate of Clinical Emergency Medicine. These teaching responsibilities – together with the growing importance in Switzerland of the specialty of emergency medicine [16] and the Certificate of Clinical Emergency Medicine – have made it mandatory to develop and implement a feasible curriculum in clinical emergency medicine. 

#### Step 2: Needs assessment

Literature research led to the conclusion that the curricula of SGNOR and of the European Society of Emergency Medicine (EUSEM) are relevant for our local setting, as these provide the basis for the learning goals for the examination needed to obtain the Swiss Certificate of Clinical Emergency Medicine. In addition to these content frameworks, simulation-based emergency medicine curricula were identified and these helped with the choice of teaching formats included in our curriculum [17], [18]. Targeted needs assessment of all physicians working in the ED revealed the local learning needs that are specific for our local setting, e.g. specific knowledge about sedation procedures and ventilation techniques, as well as the preferred learning methods of the local team. Our needs assessment of ED physicians favoured knowledge transfer by interactive simulation-based team training sessions, manual skill training workshops, and time-independent, video recorded lectures. This is in accordance with the needs assessment of Shappell et al. [10]. 

As a third step, the curricula of SGNOR and EUSEM were assessed in detail by all 28 local physicians working at our ED. A workshop was conducted to tailor the content to local needs, as identified through the targeted needs assessment conducted by all physicians in the second part. We focused on topics with either high prevalence or relevance in our ED setting. For example, one topic covered the differential diagnosis of chest pain including myocardial infarction. This is of high prevalence, in contrast to aortic dissection or the Boerhaave syndrome, which is highly relevant, but less prevalent. Other major areas covered are for example “shock”, “approach to patients with dyspnoea”, “sepsis”, or “electrolytic disturbances”. A detailed list of topics is available from the corresponding author on request.

#### Steps 3 and 4: Definition of learning goals and allocation of educational strategies

The topics identified in the curricular workshop adapted from the EUSEM and SGNOR outcome frameworks [2], [3] are integrated into the curriculum according to the learning modality identified as being most appropriate. Annual activities in our ED on the emergency medicine curriculum are summarised in Table 1 (Tab. 1). 

#### Step 5: Implementation

To achieve standardisation, a mandatory blueprint was defined for the implementation of all teaching activities (available upon request from the authors).

Knowledge of the basics of emergency medicine is transferred in weekly lectures. 

The lectures are recorded and are available online, independent of time and location, to all physicians and nurses of our department – after entering a secure password-protected video platform. 6 months after starting podcast recording, about 30 videos with a combined playback time of 12 hours and about 450 views from about 30 doctors were uploaded and available online. After one year, the recurrent topics are checked and updated or supplemented as appropriate. Topics not covered by traditional lectures, e.g. examination techniques or faculty development initiatives on feedback and mentoring, are taught in extended curriculum workshops. Simulation-based team training sessions for the trauma room as well as combined learning workshops on procedure – for e.g. analgosedation and non-invasive ventilation – are all covered by interdisciplinary and interprofessional teaching. Interprofessional and interdisciplinary training in analgosedation was developed and implemented, and a clear effect on clinical outcome could then be demonstrated [15]. All simulation-based teaching sessions are based on the framework for high fidelity medical simulations, in order to achieve effective learning, as in Issenberg et al [19]. All simulation sessions integrated in our curriculum focus intensely on providing feedback, integrate multiple learning strategies and are conducted in a controlled environment. This ensures that students are provided with a secure learning opportunity, where they can make and correct errors. The simulation-based training sessions aim to establish a culture of safety by teaching simulation-based team work, as we agree with Croskerry et al. that a safety culture cannot be taught by reading or lectures [20].

#### Step 6: Evaluation with follow-up survey 6 months after implementation

The response rate to the pre-curriculum questionnaire was 12 (43%) vs. 24 (86%) in the follow-up survey. The pre-curriculum survey showed good acceptance of the simulation-based training sessions, as well as of the skill training sessions already implemented in our department (see Table 2 (Tab. 2)). After the curriculum had been implemented, a follow-up survey was performed. This confirmed that the simulation-based training sessions were accepted (in accordance with the initial evaluation), as were the skill training sessions and the new curricular components (see Table 2 (Tab. 2)). The participants in the evaluation preferred the hands-on teaching formats (simulation-based training formats and skill training) to the lecture-based teaching formats.

As surrogate parameter for overall satisfaction with the learning opportunities in the department, physicians were asked before and after the implementation, if they were satisfied with the learning experience in their working environment. In comparison with the pre-curriculum survey, there was an increase in satisfaction with learning during work (number satisfied pre-implementation 5 (50%) vs. post-implementation 19 (95%)) 

## Discussion

Outcome based education is the current gold standard in medical education [6]. Although many outcome frameworks for both undergraduate and postgraduate education have been developed [5], the devil is often in the details of their implementation [21], [22], [23]. This problem is no different in emergency medicine, where outcome frameworks have been developed [3], but reports of successful implementation are lacking. 

In disciplines requiring trainees to work in shifts, the best format to choose may be time- and location-independent online and blended learning. This should be accompanied by focused team-training sessions that are interprofessional, interdisciplinary and simulation-based (in-situ). Hayden et al. [24] have recently stressed the importance of integrating the teaching of human factors with simulation-based education, in order to improve the interface between technologies and individuals in emergency medicine. Careful alignment of objectives to needs and formats is monitored at multiple time points during all phases of curriculum development at our ED. This is not only required for reasons of public accountability [21], but is also a necessity for theoretical reasons, as Biggs and Tang summarised in their “constructive alignment concept” [25]. Furthermore, Locke et al. demonstrated that learner satisfaction and performance are enhanced if there is a clear goal with which trainees can identify [26]. 

While such an alignment requires considerable effort, the results of our approach justify the extra mile: Using Kirkpatrick`s model [27] for summative evaluation, our survey of physicians at our department showed increased overall satisfaction with the learning opportunities provided. At the level of learning outcomes, we evaluated the knowledge gain and self-efficacy in the participants in our procedural training sessions [15]. For one component of our curriculum, interprofessional and interdisciplinary sedation, the improvement in patient care has been demonstrated at the highest level, i.e. patient/health outcomes [15].

While the specific results of our alignment may only be of interest to university staff who are responsible and interested in the education of emergency physicians, the general approach may be useful in other disciplines facing similar challenges, such as shift work, staff rotation and limited staff availability on site.

### Lesson learned

After the first months of implementation of the podcasted lectures, the number of podcast views was unfortunately rather low. The feedback discussions with the learners concluded that this limited acceptance was mainly because the podcasts were only available at the place of work. It therefore became clear that access to the videos should not only be independent of time, but also of location. Because of this requirement, our podcast video platform was transferred from a local internal hospital website to a protected web-based site (https://tube.switch.ch/). If podcasts and other educational resources are available online, this may be useful for smaller hospitals with limited resources, assuming that the staff wish to participate in a formal emergency medicine curriculum within a larger area network. Educators working in highly interdisciplinary environments should also consider that not all learners may have a need for all education in their setting. In the example of an ED, an experienced internal physician may very well be interested in learning about intraosseous access, a topic that is hardly new to his anaesthetist colleague, but may skip the module on acute coronary syndrome, which is thoroughly familiar.

To further improve attendance, SGNOR credits are available for all the different formats, as attendance is significantly increased at lectures offering continuing medical education credits [28]. 

During the implementation phase of the curriculum, the importance of faculty development and capacity building became apparent, especially for the conduction of time- and resource-intensive training formats, such as training sessions for high fidelity teams and the delivery of sonography training according to the requirements of the Swiss Society of Ultrasound in Medicine (SGUM). Our curriculum in the present form may only be a starting point in the fast evolving speciality of emergency medicine in Switzerland. Future developments, such as a master program in emergency medicine, might further improve the speciality of emergency medicine.

Another critical point during implementation was the issue of quality assurance for all curricular activities. To maintain the highest quality, all our instructors conducting simulation-based trainings are trained experts in simulation-based education. In addition, our department supports postgraduate training for physicians. As a result, all educational activities are supervised by educational specialists at the postgraduate master level (Master of Medical Education, University of Bern).

#### Limitations

Meaningful outcomes of the implementation of outcome frameworks are notoriously difficult to measure, especially at higher levels of Kirkpatrick’s model for summative evaluation [27]. The work described above is no different in that regard. While we have previously demonstrated the effect on patient outcome for a single educational intervention contained in the curriculum described [15], the effect of any educational intervention – let alone a whole curriculum – is often diluted by the many other factors that affect patient outcome [29], [30]. This manuscript is a project description and aims to share insights and experience with the development and implementation of an ED curriculum. Therefore data presented about the evaluation and data for outcomes are limited – especially on the higher levels of Kirkpatrick`s model. Further research is necessary to confirm the success of this curriculum model – with detailed evaluation, including hard outcomes. 

The identified learning fields and specific topics as well as allocated teaching formats are tailored to our specific setting in an emergency department and may not necessarily be transferable without adaption to another setting. Nevertheless, our curriculum is based not only on national requirements defined by SGNOR but also on EUSEM objectives, and thus may serve as one possible implementation; it should be feasible to transfer and adapt this approach to other emergency departments in Switzerland or elsewhere in Europe.

## Conclusions

The combination of multiple teaching formats, from time- and location-independent solutions as podcasted lectures to simulation-based training sessions, as well as small group workshops and skill training sessions, might be a valuable possibility to implement a state-of-the-art emergency medicine curriculum in a busy emergency department. This development and implementation may serve as an example in the fast evolving speciality of emergency medicine and can demonstrate solutions - not only for the university setting but also for smaller hospitals with limited resources. As the implementation of a whole curriculum is protracted and requires a great deal of work, collaboration between emergency departments is highly desirable and our time- and location-independent activities are ideally suitable for a collaborative curriculum project.

## Abbreviations

- ED – emergency department
- SGNOR – Swiss Society of Emergency Medicine
- EUSEM – European Society of Emergency Medicine
- SGUM – Swiss Society of Ultrasound in Medicine

## Acknowledgement

The authors want to thank the anonymous reviewers for their critical review of our manuscript.

## Competing interests

The authors declare that they have no competing interests. 

## Figures and Tables

**Table 1:**
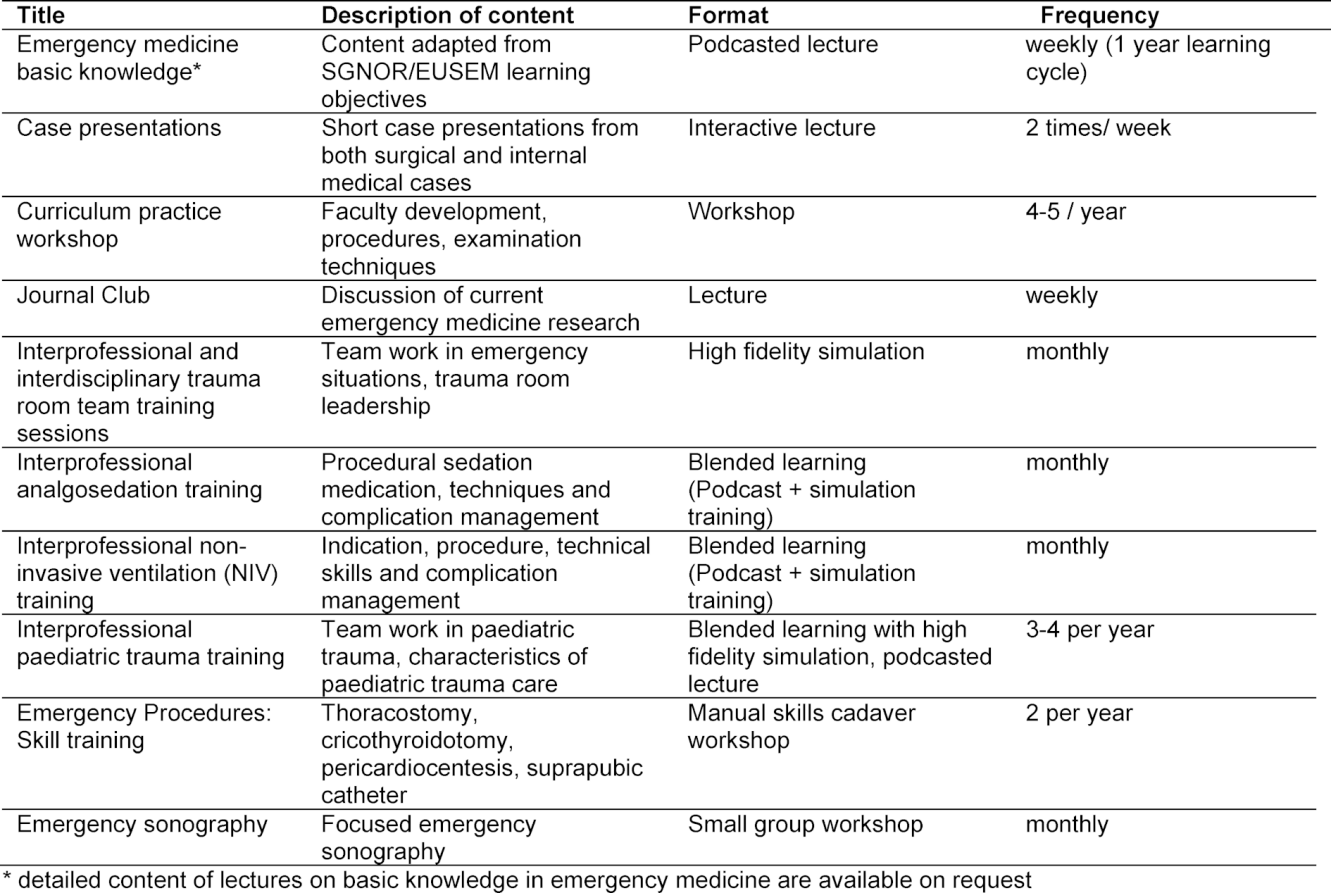
Summary of curricular teaching activities

**Table 2:**
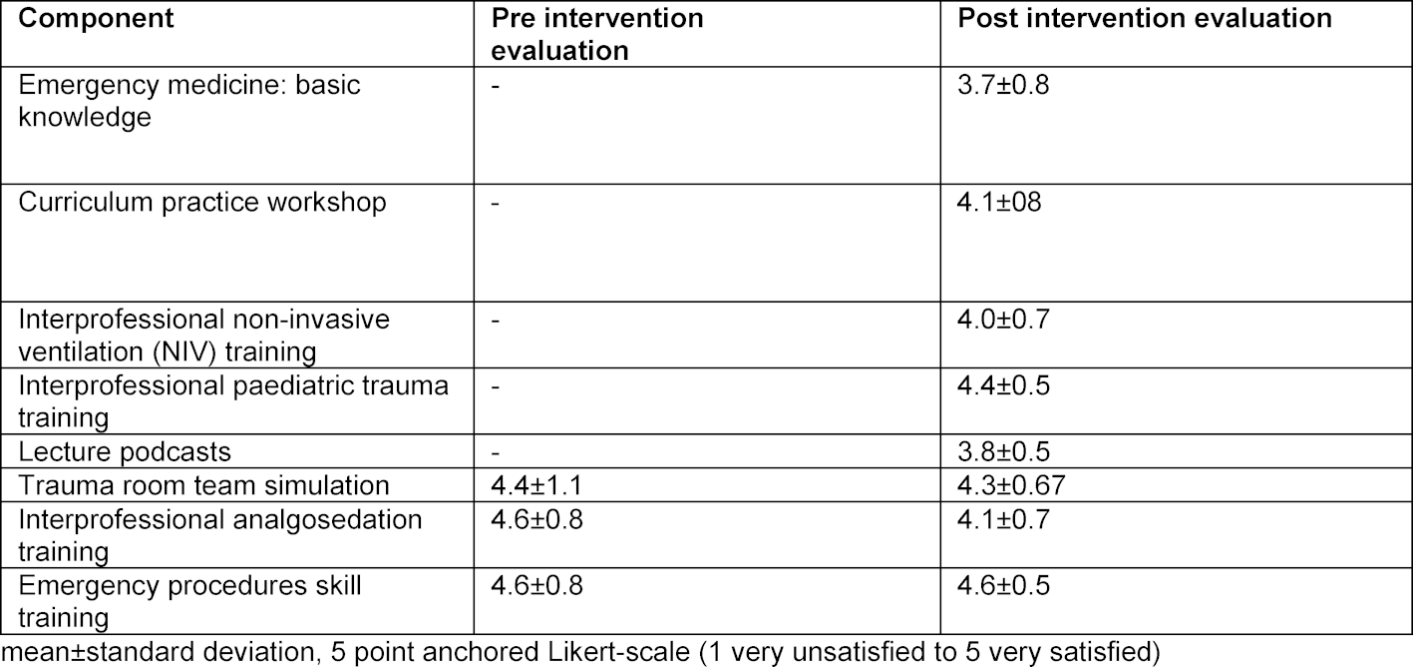
Evaluation of new curricular components
